# Amino Acid (Leucine) Chromatography: A Study of Branched-Chain Aminoaciduria in Type 2 Diabetes

**DOI:** 10.7759/cureus.1091

**Published:** 2017-03-12

**Authors:** Bhagavan Reddy Kolanu, Venugopal Boddula, Sabitha Vadakedath, Venkataramana Kandi

**Affiliations:** 1 Biochemistry, Prathima Institute of Medical Sciences; 2 Biochemistry, Chalmeda Anand Rao Institute of Medical Sciences; 3 Department of Microbiology, Prathima Institute of Medical Sciences

**Keywords:** leucine, thin-layer chromatography (tlc), mammalian target of rapamycin (mtor), branched chain aminoacids (bcaa's), type 2 diabetes (t2d)

## Abstract

**Introduction:**

Diabetes is a disease characterized by insulin deficiency resulting in glucose intolerance and in abnormalities of other metabolic fuels including protein. Recently, a number of studies have revealed that branched-chain amino acids (BCAAs) (leucine, isoleucine, and valine) play an important role in the regulation of protein synthesis by activating mammalian target of rapamycin (mTOR) in pancreatic β cells. BCAAs have positive effects on the regulation of glucose homeostasis. Leucine is an important nutrient signal as evidenced by recent observations, which showed increased fasting concentrations of circulating BCAAs being associated with an increased risk of type 2 diabetes (T2D) and insulin resistance in humans. Leucine seems to have direct effects on hypothalamic and brainstem functioning involved in satiety, which can potentially contribute to obesity and T2D. A number of observational studies indicate that elevated activity of BCAAs could be associated with poor metabolic health and T2D complications. Although these associations were consistently observed in humans, the mechanisms underlying this relationship remain to be completely understood. In this study, we have attempted to evaluate urinary excretion of leucine among patients of T2D and compared them with healthy controls by using a low-cost and non-invasive amino acid chromatography technique.

**Methods:**

The study was carried out in the Department of Biochemistry, Central Research Unit, Prathima Institute of Medical Sciences (PIMS), Karimnagar, Telangana, India, during the period between July and September 2016. A group of 55 normal healthy subjects (control group A), and 55 patients suffering from T2D on treatment (test group B), were enrolled in the study. The urine samples were collected from normal and T2D subjects. Thin-layer chromatography (TLC) for leucine was performed on all the urine samples.

**Results:**

A strong correlation (p=0.0004) was found between the urinary excretion of leucine among the control (Rf=0.174 ±0.089) and T2D (Rf=0.247 ±0.030) patients.

**Conclusion:**

Excretion of BCAAs (leucine) in detectable and increased quantities reflect the presence of an altered metabolic state attributable to T2D, which in turn could lead to early diabetic complications. This method (TLC), being non-invasive and cost-effective, could be recommended for assessing the progression and management of type 2 diabetes patients.

## Introduction

The prevalence of type 2 diabetes (T2D) is increasing worldwide, as evidenced from the fact that more than 90% of individuals suffer from T2D compared to type 1 diabetes. The major feature of obesity and T2D is a metabolic syndrome which increases the risk of coronary heart diseases, cancer, neurodegenerative disorders, and other metabolic abnormalities [1]. Sedentary lifestyles and dietary habits account for the increased rates of diabetes in India. Food habits in India have changed from traditional to processed foods with more calories, more oil, ghee, and dalda, and less protein, which again is a risk factor for T2D [2]. We know the role played by proteins and amino acids in regulating cell growth and proliferation. Leucine is one of the essential branched-chain amino acids (BCAAs), along with valine and isoleucine, having a role in insulin secretion, regulating protein turnover, and protein synthesis [3-4]. The enzyme branched-chain amino acid transaminase (BCAT) is the one which transaminates the BCAAs to corresponding alpha keto acids. There are two forms of BCAT enzymes, one present in mitochondria, which is active in multiple tissues, but absent in the liver and gut. The cytosolic form active in the brain and peripheral tissues makes the BCAAs bypass porto-venous circulation. Leucine is an important nutrient sensor along with other BCAAs, as noted by their increased fasting levels indicating a risk factor for T2D, insulin resistance, and other metabolic diseases. Leucine has a direct effect on hypothalamic and brainstem processes involved in satiety [5] and can regulate the release of hormones like leptin and ghrelin that can potentially affect food intake and glucose levels [6-7]. The present study aims to know the levels of leucine excreted in urine by T2D patients when compared to controls.

## Materials and methods

The study group included 55 patients having T2D attending the outpatient ward of Prathima Institute of Medical Sciences, Karimnagar, Telangana, and 55 normal healthy controls, in the age group of 30-65 years. The study was approved by the institutional ethical committee. Urine samples of all subjects were collected for analysis after obtaining informed consent. In all the urine samples, the migration of leucine amino acid was carried out using thin-layer chromatography (TLC).

### Thin-layer chromatography (TLC)

TLC is a quick, inexpensive microscale technique used to measure amino acid migration, which is calculated as retardation factor (Rf).

TLC was standardized in our laboratory by using standard amino acids. In the present study, ready-made TLC plates were used for analysis. The solvent used here constituted a mixture of butanol, acetic acid, and water in the ratio of 4:1:5. The standard amino acid used for comparing the migration of amino acid is a 2% solution of leucine.

**Figure 1:**
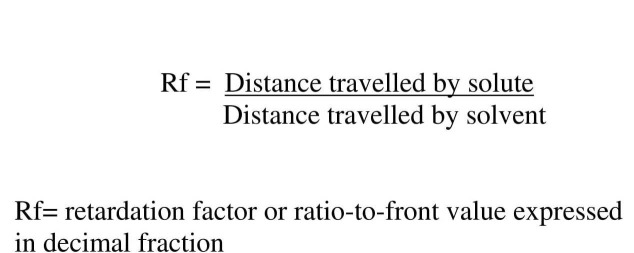
Formula to calculate the retardation factor (Rf) value

A small amount of the urine sample (approximately two drops) was dispensed with a capillary tube on a TLC plate and was allowed to dry. The plate was then carefully placed in a trough containing the solvent mixture (butanol, acetic acid, and water at a ratio of 4:1:5). The watery component of the solvent mixture binds to the inert support on the plate and serves as a stationary phase. The sample component, which migrates on the plate, is considered as the mobile phase. Separation occurs due to the difference in the solubilities of the mobile and stationary phases. The whole process of separation took around two hours, after which the TLC plate was dried and later sprayed with a coloring reagent (ninhydrin) that helps in visualization of migration. The length of migration was simultaneously compared with standards, and the corresponding Rf values were calculated using the formula as shown in Figure 1.

## Results

The results of TLC was recorded as retardation factor (Rf) value. A ‘p’ value of 0.0004 was observed between the two groups, revealing statistical significance as shown in Table 1.

**Table 1:** Comparision of thin-layer chromatography of leucine among control and test groups

Thin-layer chromatography (TLC)	Group-A (Control) n=25 Mean±SD	Group-B (Type 2-DM) n=25 Mean±SD	‘p’ VALUE
Rf Value	0.174±0.089	0.247±0.030	0.0004*

## Discussion

In T2D, there is either progressive degeneration of pancreatic islet cells or receptor defect for insulin. Increased rates of proliferation of pancreatic β cells as well as apoptosis were also noted in diabetes [8]. Leucine stimulates β cell proliferation via nutrient signaling, activates Rag guanosine triphosphatases (GTPases), activates mammalian target of rapamycin complex 1 (mTORC1), and increases translational protein activity, which forms a hallmark feature of T2D as evidenced from studies in experimental mice [9]. Leucine can also trigger triglyceride synthesis in the liver by activating sterol regulatory element binding protein (SREBP) and overactivating folding of newly synthesized protein in the endoplasmic reticulum (ER), thus causing β cell dysfunction and death, which can lead to T2D [10].

In obesity, the uptake of BCAAs by adipose tissue is less when compared to skeletal muscle. In obesity, there is muscular atrophy due to increased cortisol levels due to changes in Kruppel-like factor 15 (KLF15) in skeletal muscle. The KLF 15 upregulates gene expression of branched-chain aminotransferase 2 (BCAT 2), which could later contribute to insulin resistance [11-12].

### Role of leucine in insulin secretion

In pancreatic β cells, leucine simulates the release of insulin and provides amino group for increased transportation across the membrane by means of activating the glutamate dehydrogenase (GLDH) enzyme. Leucine regulates gene transcription and protein synthesis in pancreatic β cells via mammalian target of rapamycin (mTOR) signaling, or it can directly incorporate ribosomes for protein synthesis [13].

### mTOR dependent signaling of leucine

Leucine is one of the nutrient sensors regulating the mTOR protein complex. mTOR is a multi-domain protein; in mammals, there are two complexes - mTORC1 and mTORC2 respectively. mTORC1 consists of mTOR, regulatory-associated protein of mTOR (raptor), G protein beta subunit-like (GβL), and DEP domain-containing mTOR-interacting protein (DEPTOR). Its function is to regulate cell growth and proliferation. mTORC2 is made of mTOR, rapamycin-insensitive companion of mammalian target of rapamycin (Rictor), GβL, stress-activated protein kinase-interacting 1 (Sin 1), proline-rich protein 5 (PRR5), and DEPTOR protein; it promotes cellular survival, regulates cytoskeletal dynamics, and controls ion transport as well as cell growth [14-15]. There is aberrant signaling of mTOR during conditions like cancer, cardiovascular disease (CVD), and diabetes.

**Figure 2:**
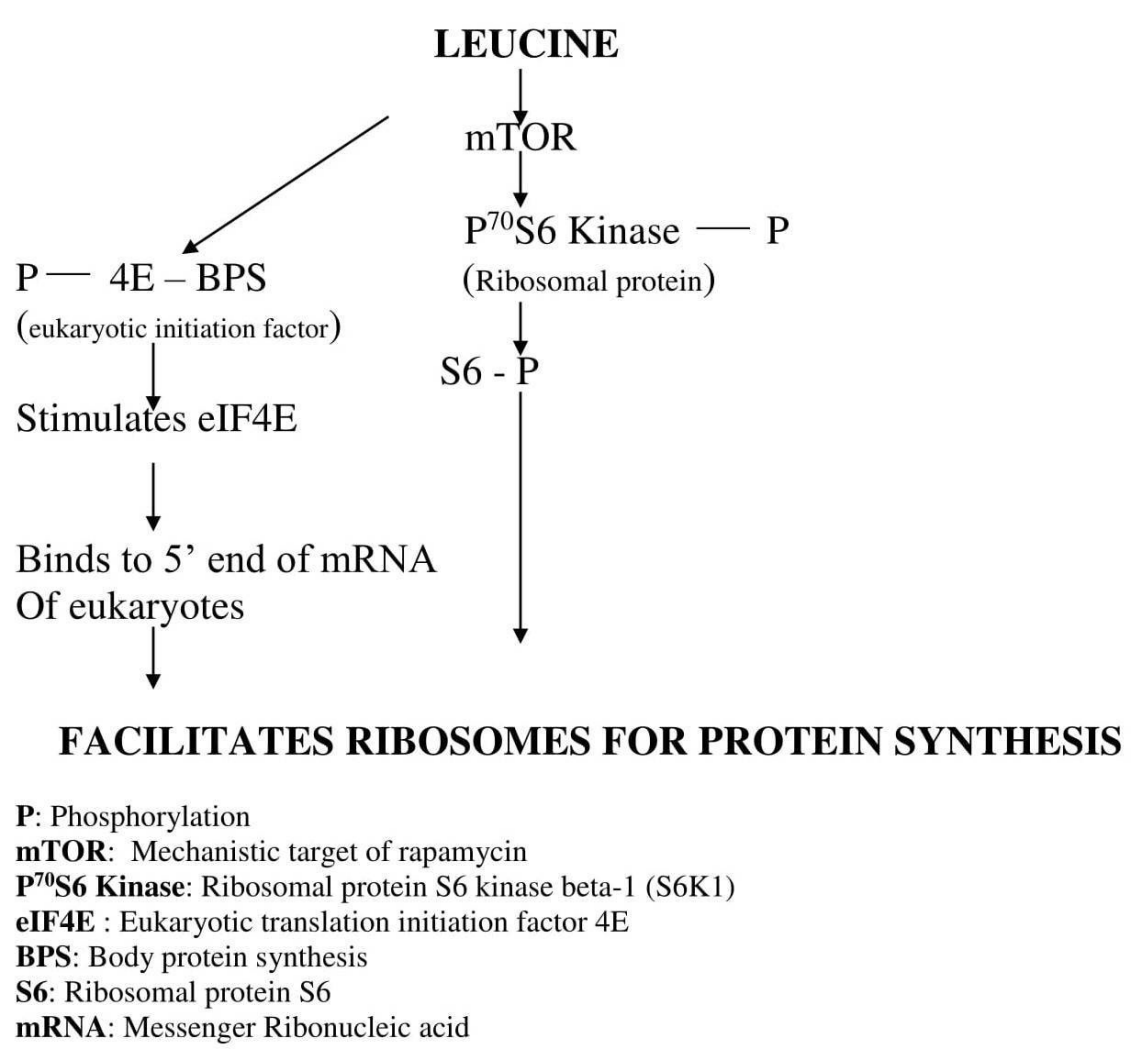
mTOR dependent protein synthesis by leucine

In this pathway, leucine inhibits adenosine mono phosphate protein kinase (AMPK) because of the energy generated in the muscle cells by it, and there is phosphorylation of eukaryotic translation initiation factor 4 G protein (eIF4G) and ribosomal protein P^70^S6 kinase as shown in Figure 2. All these events lead to recruiting ribosomes for protein synthesis [16-18].

 

### mTOR independent signaling by Leucine

**Figure 3:**
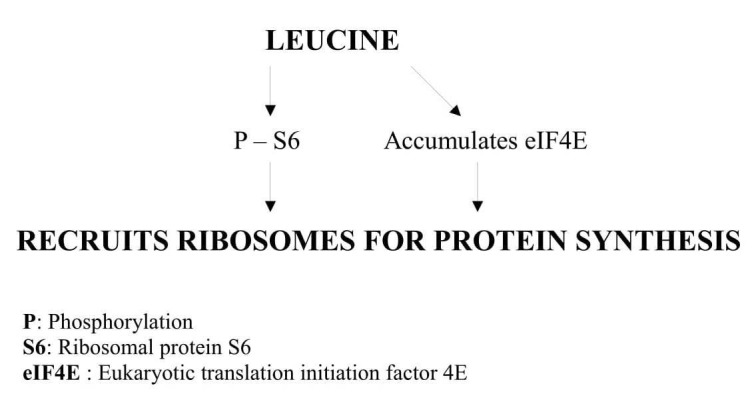
mTOR independent protein synthesis by leucine

Leucine can initiate protein synthesis without mTOR association and can synthesize protein as shown in Figure 3.

It was found in experimental animals that leucine alone can phosphorylate serine residues at 1108, 1148, and 1192 of C-terminal end of eIF4G, forming a complex of eIF4G and eukaryotic translation initiation factor 4E (eIF4E). Thus, it initiates protein synthesis by translation, especially in skeletal muscles irrespective of fiber type [19-20].

Many previous studies have reported the role of circulating and dietary BCAAs and their potential role in the development and progression of various diseases including T2D [21-25]. This is probably the first report which compared the excretion of leucine in urine among T2D patients with the controls negative for T2D. Detection of leucine in blood circulation is a rather invasive and costly method which can be replaced by studying urinary excretion of BCAAs.

## Conclusions

Diagnosis and management of patients suffering from T2D assume greater significance, because if it is not controlled, the patients may suffer from various complications including chronic kidney diseases, cardiovascular disease, neurological disease, and other metabolic disorders.The study results have emphasized the probable role of BCAAs and their excretion on the progression of T2D. Future studies are warranted to investigate the importance of BCAAs, and their supplementation in the management of various metabolic disorders including T2D. Leucine, including other BCAAs and the genes coding for their catabolism, could well be potential biomarkers for the diagnosis and management of various metabolic disorders.
